# Cortical entrainment to speech produced by cochlear implant talkers and normal-hearing talkers

**DOI:** 10.3389/fnins.2022.927872

**Published:** 2022-08-09

**Authors:** Shruthi Raghavendra, Sungmin Lee, Hyungi Chun, Brett A. Martin, Chin-Tuan Tan

**Affiliations:** ^1^Department of Electrical and Computer Engineering, University of Texas at Dallas, Richardson, TX, United States; ^2^Department of Speech-Language Pathology and Audiology, Tongmyong University, Busan, South Korea; ^3^Graduate Center, City University of New York, New York City, NY, United States

**Keywords:** cortical entrainment, electroencephalogram, cochlear implant, perceived sound quality, speech envelope

## Abstract

Cochlear implants (CIs) are commonly used to restore the ability to hear in those with severe or profound hearing loss. CIs provide the necessary auditory feedback for them to monitor and control speech production. However, the speech produced by CI users may not be fully restored to achieve similar perceived sound quality to that produced by normal-hearing talkers and this difference is easily noticeable in their daily conversation. In this study, we attempt to address this difference as perceived by normal-hearing listeners, when listening to continuous speech produced by CI talkers and normal-hearing talkers. We used a regenerative model to decode and reconstruct the speech envelope from the single-trial electroencephalogram (EEG) recorded on the scalp of the normal-hearing listeners. Bootstrap Spearman correlation between the actual speech envelope and the envelope reconstructed from the EEG was computed as a metric to quantify the difference in response to the speech produced by the two talker groups. The same listeners were asked to rate the perceived sound quality of the speech produced by the two talker groups as a behavioral sound quality assessment. The results show that both the perceived sound quality ratings and the computed metric, which can be seen as the degree of cortical entrainment to the actual speech envelope across the normal-hearing listeners, were higher in value for speech produced by normal hearing talkers than that for CI talkers. The first purpose of the study was to determine how well the envelope of speech is represented neurophysiologically via its similarity to the envelope reconstructed from EEG. The second purpose was to show how well this representation of speech for both CI and normal hearing talker groups differentiates in term of perceived sound quality.

## Introduction

Sound quality is classically estimated from the physical difference of an utterance produced by a talker from its standard reference and is used as a metric to quantify “how well” the talker has spoken (Loizou, 2011), which may not align well with outcomes obtained perceptually. Perceived sound quality-based listener judgments may be a more direct way to determine “how well” talker has spoken, however, the outcome can vary greatly from one listener to another. Adding physiological data measurement (i.e., cortical activity) to the behavioral sound quality judgments may facilitate the needed consistency and consensus across listeners.

A cochlear implant (CI) is a common device used to restore the ability to hear and provide the necessary auditory feedback to produce and monitor speech for hard-of-hearing individuals. However, there remains a large variability in speech production proficiency among implant recipients, which could be attributed to the age of implantation, duration of hearing loss, duration of device used and remaining residual hearing (Ruff et al., 2017; Kim et al., 2018; Gautam et al., 2019). In this study, we obtained perceived sound quality ratings of speech produced by both a CI talker group and a normal-hearing (NH) talker group and captured their cortical entrainment to the speech as indicated by associated cortical activities of the normal-listening listeners. The first purpose of the study was to determine how well the actual envelope of speech is represented neurophysiologically via its similarity to the envelope reconstructed from the co-fluctuating electroencephalogram (EEG) activities using a re-generative model (Crosse et al., 2016). The second purpose was to show how well the speech envelope was represented in response to both CI and NH talker groups and differentiated the groups in terms of their perceived sound quality. The goal is to achieve a metric to assess “how well” hard-of-hearing talkers have spoken and the auditory feedback they received in their current aural compensation.

Neurophysiological processing of sound is usually examined using event-related potentials (ERPs). The P3 component has been recently used to evaluate the effects of perceived quality changes in speech (Uhrig et al., 2019a,b) and it was reported that the peak amplitude and latency were modulated by sound quality. Likewise, others studies (Martin et al., 1997; Martin and Stapells, 2005; Antons et al., 2010, 2012; Porbadnigk et al., 2013) also showed that, as the level of degradation decreases when compared to the standard stimulus, which means it is harder for listeners to discriminate the deviant stimulus from the standard stimulus, the amplitude and latency of the P3 component become lower and longer, respectively. These ERP techniques commonly utilize auditory stimuli of short duration, which are not optimal to use to make perceptual sound quality judgments.

Cortical entrainment to the envelope of speech may serve as a useful alternative to investigate the neurophysiologic processing of continuous speech, as evidence has shown that the dynamic cortical activity tracks the envelope of continuous, natural speech (Aiken and Picton, 2008; Lalor and Foxe, 2010). This phenomenon reflects the activity of distinct neural populations that implement different functional roles including encoding acoustic features (for a review, see Ding and Simon, 2014) and can be captured by EEG (Aiken and Picton, 2008). The temporal envelope, a slow variation of the amplitude of speech is considered to be one of the most important cues for speech intelligibility (Peelle and Davis, 2012) and speech perception (Shannon et al., 1995). Particularly in delta (1–4 Hz) and theta (4–8 Hz) frequency bands, neural activity is known to track the amplitude envelope of speech (Ding and Simon, 2014).

This cortical tracking of the speech envelope can be inferred from the correlation between the actual speech envelope and the speech envelope predicted/decoded from the EEG/magnetoencephalography (MEG). Many studies have demonstrated that the speech envelope can be decoded from single-trial EEG/MEG recordings obtained by presenting the stimulus only once (Ding and Simon, 2012, 2013; Di Liberto et al., 2015; O’Sullivan et al., 2015). A multivariate linear model (Crosse et al., 2016) was developed to map the multi-channel EEG signal into a single-channel speech envelope with the intent of minimizing the mean-squared error between the actual speech envelope and the reconstructed envelope. To accomplish this, the time-shifted version of the EEG channels is first obtained by applying a range of delays also known as the temporal integration window (e.g., 0 and 500 ms) to each channel, then all of the delayed channels are weighted, to linearly reconstruct the envelope of speech. The actual speech envelope and the reconstructed envelope are then correlated with each other, which yields a measure of envelope entrainment. Using this technique, previous studies have examined the cortical entrainment to the envelope of speech and correlated the degree of entrainment to behavioral speech intelligibility (Ding and Simon, 2013; Kong et al., 2015; Vanthornhout et al., 2018). It has been shown that higher speech intelligibility coincides with improved cortical entrainment to the speech envelope. This technique was also used as an EEG-based measure of attention decoding in a cocktail party environment (O’Sullivan et al., 2015).

Likewise, we used this technique to study the cortical entrainment to the speech envelope in relation to sound quality as perceived by normal-hearing listeners, and developed a metric to differentiate speech spoken by CI and NH talker groups. Bootstrapped Spearman correlation between the actual speech envelope and the envelope reconstructed from the EEG was computed to quantify the cortical entrainment to the speech envelope, and compared to the sound quality as perceived by the listener. We hypothesized there would be closer cortical tracking of the speech envelope (higher correlation) when speech is of higher perceived sound quality. We therefore anticipated that closer cortical tracking of speech envelope would be obtained with speech passages spoken by NH talkers than with those spoken by CI talkers.

## Materials

### Participants

Eleven normal-hearing listeners were recruited from the University of Texas at Dallas student population for this study. Their age ranged from 19 to 29 years (mean age = 21.5 years; 5 female, 6 male). All normal-hearing listeners were screened by presenting pure tones at 20 dB HL from 250 HZ to 8 kHz at octave frequencies and had normal hearing thresholds < 20 dB HL. This study was approved by the Institutional Review Board at the University of Texas at Dallas. All participants signed the informed consent forms prior to participation in the experiment and were paid for their participation.

### Talkers

Two groups of 8 talkers each were selected from the “Corpus of deaf speech for acoustic and speech production research” database collected at the University of Memphis (Mendel et al., 2017). This corpus is a pool of speech recordings digitally sampled at 44,100 Hz, spoken by NH talkers and hearing impaired (HI) talkers. The entire “Rainbow Passage” spoken by each talker was recorded by the authors using a Shure SM93 prolog dynamic microphone. We selected speech passages read by 8 CI talkers (4 female; 4 male) and 8 NH talkers (6 female; 2 male) for our present study. The two groups of talkers are, respectively, referred to as CI talker group and NH talker group. Table 1 shows the duration of speech passage recorded by each talker in both talker groups with mean and standard deviation across each talker group.

**TABLE 1:** Duration of the stimuli.

CI talker	#1	#2	#3	#4	#5	#6	#7	#8	$\bar{x}$	s.d
Duration of spoken passage (s)	115	105	115	136	127	108	156	141	125.4	17.8

**NH talker**	**#1**	**#2**	**#3**	**#4**	**#5**	**#6**	**#7**	**#8**	** $\bar{x}$ **	**s.d**

Duration of spoken passage (s)	78	94	88	88	87	91	104	83	89.1	7.7

Table 2 presents the demographic details of the chosen CI talkers from the database (Mendel et al., 2017). The CI talkers are aged between 16 and 77 years with an average age of 47.5 years. Other than CI talker #3, the rest of the CI talkers were post-lingually deaf. This should not be confused with the participants in our study who are normal-hearing listeners listening to the speech passages produced by these CI talkers and NH talkers.

**TABLE 2:** Demographic details of the CI talkers from the deaf speech corpus.

CI talker	Age (years)	Gender	Age of first amplification use (years)		Onset of hearing loss	Current type of amplification		Communication mode^4^
			Right	Left		Right	Left	
#1	37	Male	5	5	Post-lingual	CI^2^	CI	Oral
#2	38	Female	28	NA^1^	Post-lingual	CI	NA	Oral
#3	16	Female	0.5	2	Pre-lingual	HA^3^	CI	Oral
#4	77	Male	18	73	Post-lingual	HA	CI	Oral and sign
#5	62	Female	38	38	Post-lingual	CI	HA	Oral and sign
#6	60	Male	52	52	Post-lingual	HA	CI	Oral and sign
#7	57	Female	3	58	Post-lingual	HA	CI	Oral
#8	33	Male	NA	3	Post-lingual	NA	CI	Sign only

^1^NA, not applicable. ^2^CI, cochlear implant. ^3^HA, hearing aid. ^4^Oral indicates that the talker used oral speech and language. Sign indicates that the talker used sign language.

## Methods

**FIGURE 1:**
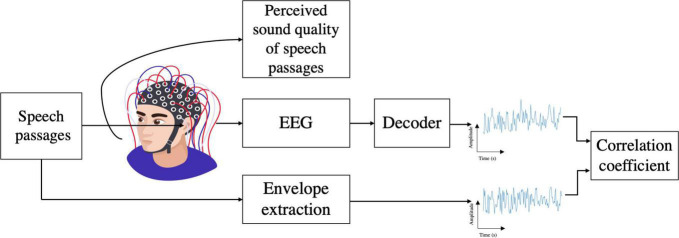
Behavioral and EEG experiment setup.

Figure 1 shows the overall setup for both behavioral and electrophysiological experiments. Normal-hearing listeners listened to the speech passages produced by both CI talkers and NH talkers. The behavioral sound quality assessment experiment was always conducted first, followed by the EEG experiment, which were performed on the same day. For each listener, the total duration of the behavioral and EEG experiments varied between 2 and 3 h. In the behavioral experiment, each listener was presented with speech passages spoken by 8 CI talkers and 8 NH talkers in a randomized order and was asked to rate the perceived sound quality of each speech passage. In the electrophysiological experiment, the single-trial EEG responses of the same listeners were recorded while they were presented with the same speech passages they heard in the behavioral experiment. The stimulus presentation order was randomized across the two experiments.

From each speech passage, the envelope was extracted and referred as the actual speech envelope. Then the envelope of the speech was reconstructed from its associated EEG signal using a decoder referred to as the reconstructed/predicted envelope. The bootstrapped Spearman correlation between the actual speech envelope and the reconstructed envelope is employed as a metric that measures the cortical entrainment to the actual speech envelope in each normal-hearing listener. Then the sound quality and the cortical entrainment to the speech envelope were compared for the speech passages produced by two groups of talkers.

### Behavioral experiment

For the sound quality assessment, listeners were seated in a soundproof booth in front of a touch screen computer monitor. They were seated 1 m from a loudspeaker at 0°azimuth at their ear height. The speech passages were presented via the loudspeaker at 65 dB SPL. Each normal-hearing listener was asked to perform two trials of behavioral sound quality assessments. In one trial, the speech passages spoken by 8 CI talkers and 8 NH talkers (total of 16 passages) were presented one at a time in a randomized order to a listener. Each speech passage was presented only one time. Listeners were instructed to listen to the speech passages and to rate the sound quality of each passage on a Likert 10 point scale (Sangthong, 2020), with 1 being the most distorted and 10 being the most undistorted using a touch screen monitor. In the second trial, the same speech passages were randomly presented and listeners again rated the sound quality. The perceived sound quality rating of each speech passage was computed as the average of the ratings obtained across the two trials.

### Electroencephalogram experiment

EEG recording was performed after the behavioral sound quality rating assessment. A 64-channel actiCHamp amplifier EEG setup (Brain Products GmbH, Munich, Germany) was used to record the ongoing EEG in response to the same passages produced by two groups of talkers used in the behavioral test. The EEG signals were recorded using an electrode cap (actiCAP, Brain Products GmbH, Munich, Germany) placed in accord to the 10–20 system (Oostenveld and Praamstra, 2001). The ground channel and the reference channel were located at FPz and FCz, respectively. To monitor eye-movement artifacts, the HEOG was monitored from electrodes placed at the lateral outer canthi and the VEOG was recorded from electrodes placed above and below the left eye. All electrode impedances were maintained below 10 kOhms. Each listener was asked to minimize body movement and watch a silent, captioned movie while EEG recording was in progress. EEG data were recorded with a sampling rate of 1,000 Hz. The EEG recordings were time aligned with the stimulus based on a trigger event inserted at the onset of each passage.

### Electroencephalogram preprocessing

All EEG data were analyzed offline using custom scripts in MATLAB_R2021a (MathWorks, Natick, MA, United States). EEG data were preprocessed using the EEGLAB toolbox (Version14.1.2b; Delorme and Makeig, 2004) in MATLAB to prune unwanted artifacts. The portion of the EEG contaminated with artifacts related to muscle was removed by visual inspection. Artifacts from the eye blink, lateral eye movement, and heart beat were pruned from the EEG using independent component analysis (ICA) in the EEGLAB toolbox. To prune the independent components reflecting eye blinks and lateral eye movements, the fully automated Eye-Catch approach (Bigdely-Shamlo et al., 2013) was used. After the artifact removal procedure, the data were re-referenced to a common average reference (CAR) and the reference channel FCz was added back to the data.

### Signal processing: Electroencephalogram and speech envelope

#### Electroencephalogram

**FIGURE 2:**
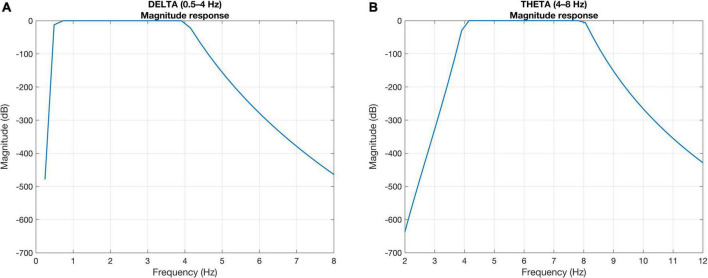
Magnitude responses of the Butterworth filter showing sharper roll-off at the edge of the pass bands in **(A)** delta and **(B)** theta frequency bands.

Cortical tracking of the acoustic features of speech is typically analyzed in specific frequency bands, including delta (0.5–4 Hz), theta (4–8 Hz), alpha (8–14 Hz), beta (14–30 Hz), low-gamma (30–70 Hz), and high-gamma (70–100 Hz). Cortical activities in delta (1–4 Hz) and theta (4–8 Hz) frequency bands are particularly known to track the amplitude envelope of speech (Ding and Simon, 2014) and are the focus here. To extract the EEG activity in the delta and theta frequency bands, the preprocessed EEG signal was band-pass filtered between 0.5–4 Hz (delta) and 4–8 Hz (theta) using a zero-phase Butterworth filter with 80 dB attenuation at 10% outside the passband (Vanthornhout et al., 2018; Lesenfants et al., 2019a,b). The zero-phase filtering was performed using the *filtfilt* command in MATLAB. Figure 2 presents the magnitude responses of the Butterworth filters in delta (Figure 2A) and theta (Figure 2B) frequency bands designed at sampling frequency = 1,000 Hz. The computed order of the Butterworth filters was 136 and 118 (68 and 59, 2nd order in cascade), respectively, to realize the delta and theta frequency bands. We chose this filter to have a sharper roll-off at the edge of the pass band as delta and theta are consecutive frequency bands. The performance of the filters was shown in Figure 2.

#### Speech envelope

The Hilbert transformation was first applied to the speech signal, to obtain the complex-valued output. The absolute value of the complex-valued output was computed which provides the instantaneous amplitude of the signal followed by low-pass filtering at 40 Hz to extract the speech envelope. In our study, we also filtered the extracted speech envelope into 0.5–4 Hz (delta) and 4–8 Hz (theta) to match the bandwidth of the EEG signals. The extracted speech envelope was resampled to 1,000 Hz to match the sampling rate of EEG signals before applying the zero-phase Butterworth filters shown in Figure 2. All speech envelopes and EEG data were further downsampled to 128 Hz (Crosse et al., 2016) to reduce the computation time.

### Speech envelope reconstruction

A linear decoder as proposed in Crosse et al. (2016) was used to predict and reconstruct the speech envelope from the associated EEG activity. The decoder acts as a spatiotemporal filter that linearly maps the EEG to the speech envelope thereby reconstructing the speech envelope estimated from the corresponding EEG response recorded when listening to the speech passages. The time-shifted version of the EEG channels was obtained by applying a range of delays (in general, between 0 and 500 ms) to each channel, then all of the delayed channels were weighted, in order to linearly reconstruct the envelope. The actual speech envelope and the reconstructed envelope were then correlated with each other, which yields a measure of cortical entrainment to the actual speech envelope. The process is explained as follows:

Given a linear decoder *g*(τ, *n*) representing the linear mapping from the EEG response, *r*(*t*, *n*), back to the stimulus envelope s(*t*), a single estimate of the stimulus envelope $\hat{s}$(t) was computed as follows:


(1) $$\hat{s}\,\left(t\right)=\Sigma_{n}\,\Sigma_{\tau }\,r\,\left(t+\tau ,n\right)\,g\,\left(\tau ,n\right)$$

with *t* ranges from 0 to T, length of the signal. τ is the integration window length and *n* is the index of the N EEG channels. The decoder *g*(τ, *n*) was derived by minimizing the mean-squared- error (MSE) between the actual stimulus envelope s(*t*), and the estimated stimulus envelope $\hat{s}$(t), i.e.,


(2) minε(t)=Σ[s(t)-s^(t)]2t

The decoder computation can be expressed using the following matrix operation:


(3) $$\text{g}={\left({\text{R}}^{T}\,\text{R}+\lambda \,\text{I}\right)}^{-1}\,{\text{R}}^{T}\,\text{s}$$

where the superscript *T* represents the transpose of a matrix, **I** is the identity matrix and λ is the ridge/regularization parameter chosen to make the decoder less prone to overfitting. **R** represents the lagged time series of EEG response matrix r, for N channels, the dimensions of matrix **R** is T × Nτ_window_, where τ_window_ = τ_max_ - τ_min_ with τ_min_ and τ_max_ represent the minimum and maximum time lags (in samples), respectively. The stimulus envelope, **s**, is a column-wise vector of length T and the resulting decoder, **g**, would be a vector of Nτ_window_ samples.

**FIGURE 3:**
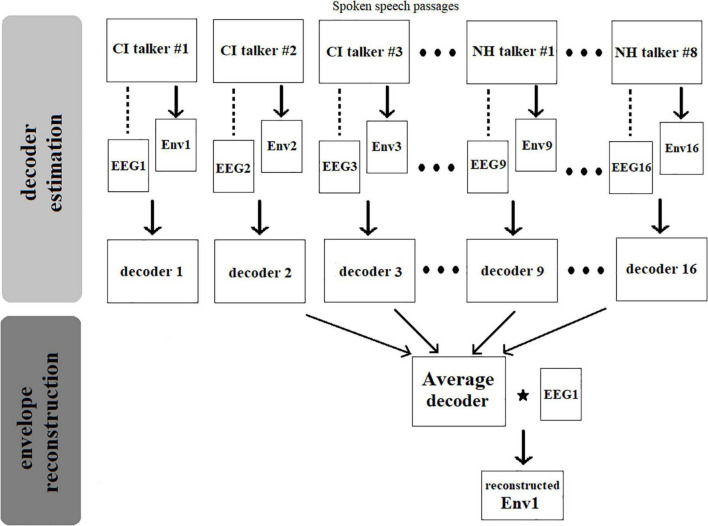
Flowchart illustrating the stages involved in the reconstruction of a speech envelope.

Figure 3 illustrates the procedure of reconstruction of a speech envelope in a single listener. For each normal-hearing listener, 16 decoders were obtained, corresponding to the 16 speech passages combined from the two groups of talkers. These 16 decoders were, respectively, computed as Eq. 3 using each of the 16 speech envelopes extracted from the 16 spoken speech passages by 8 CI and 8 NH talkers and their associated EEG signals collected from the normal-hearing listeners listening to these speech passages. Later, the reconstructed envelope which corresponds to the speech passage produced by CI talker #1 was obtained by correlating an “Average decoder” with the EEG signal recorded from the listener while listening to spoken passage by CI talker #1 as shown in Eq. 1. This “Average decoder” was computed as the average of the remaining 15 decoders, i.e., decoder 2 to decoder 16. This “leave-one-out” model approach was repeated to reconstruct the envelope corresponding to the remaining 15 speech passages in the same listener. Finally, to compute the bootstrapped Spearman correlation between the actual speech envelope and the reconstructed envelope, we randomly permuted the reconstructed envelope 1,000 times and calculated Spearman’s correlation between the result and the actual speech signal for each permutation. The final correlation value was evaluated as the averaged value of the resulted 1,000 correlation values.

The sample size for this study is supported by a power analysis conducted on the data. Cohen’s “d” (Cohen, 1988) was computed on the paired samples *t*-tests for the sound quality and envelope entrainment data. Assuming a significance level, alpha = 0.01 and power of 80%, the sample sizes were, respectively, estimated as “8” and “14” with the behavioral and EEG experimental results and our current study’s sample size falls between the estimated sample sizes.

## Results

### Behavioral results

**FIGURE 4:**
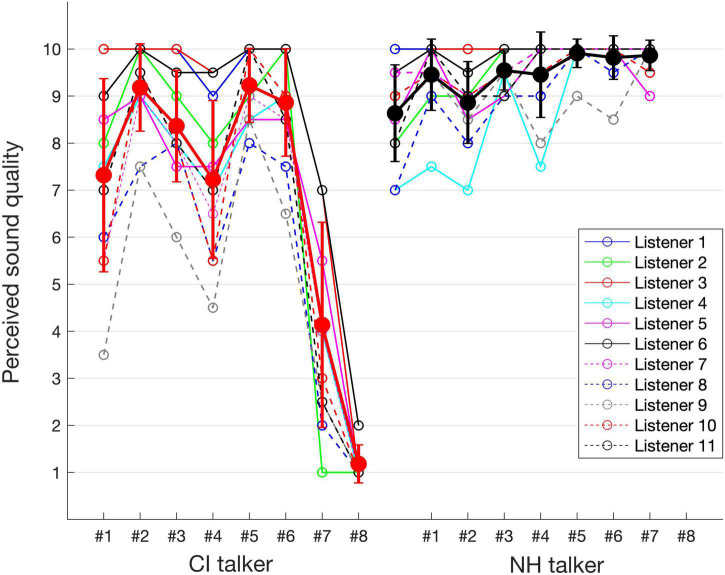
Individual listener assessed sound quality data point for the speech passages spoken by CI and NH talkers and also showing the mean and SD of the sound quality ratings for the CI and NH talker groups.

Figure 4 presents the perceived sound quality ratings as rated by individual normal-hearing listeners for the speech passages produced by the two talker groups. Figure 4 also shows the mean and standard deviation of the perceived sound quality ratings for the spoken speech passages by CI talker (red curve) and NH talker (black curve) groups across the normal-hearing listeners. The perceived sound quality ratings for the speech passages spoken by CI talkers varied widely along the range. Whereas for those spoken by the NH talkers, there was little difference in the sound quality ratings across the NH talker group as each of the speech passage spoken by NH talkers was rated almost equally high in sound quality. Within the CI talker group, CI talker #2 was rated with highest mean sound quality of 9.2 and CI talker #8 was rated with lowest mean sound quality of 1.2. In addition, there was a larger relative difference in the standard deviation for the speech passages spoken by CI talkers compared to the speech passages spoken by the NH talkers especially in case of CI talker #1, #4, and #7.

#### Statistical analysis

Statistical analysis was performed using MATLAB_R2021a and R studio (version 3.3.0). First, a paired-samples *t*-test on perceived sound quality between the CI talker group and NH talker group was performed to investigate whether there is a significant difference in perceived sound quality ratings for these two groups of talkers. For paired-samples *t*-test, the two dependent samples contained one entry for each listener with a single averaged perceived sound quality across CI talker group and NH talker group. The results showed that the mean sound quality ratings for the NH talker group were significantly higher than that of the CI talker group [*t*(10) = 9.8, *p* < 0.001]. To appropriately model our dataset statistically and to assess the relevant factors such as talker group (CI/NH), talker gender (male/female), and duration of the utterance of spoken passages (referred to as duration; Table 1) contributing to perceived sound quality, we used a linear mixed effects regression (lmer) model using the lme4 package (Bates et al., 2015). The above three fixed factors are, respectively, referred to as TALKERGROUP, TALKERGENDER, and DURATION. In the lmer model, the perceived sound quality, as rated by the listeners, was considered as the outcome variable and the listener as the random factor. By constructing two hierarchical regression models, we assessed how the three fixed factors/predictors such as TALKERGROUP, TALKERGENDER, and DURATION modulated the perceived sound quality. The hierarchical regression analysis consisted of two models: Model 1 (m1) used only TALKERGROUP as a predictor, while Model 2 (m2) used TALKERGROUP, TALKERGENDER, and DURATION as predictors. Model comparison between the simpler and the complex model, i.e., m1 and m2, respectively, was obtained with the R-function ANOVA that uses the chi-square test. The improvement in the model fit for adding more predictors was determined by comparing the Akaike Information Criterion (AIC; Akaike, 1974) of the simpler and the complex model. If the AIC of the more complex model was smaller than that of the simpler model with *p* < 0.05 (statistically significant), the more complex model was considered to have a better fit. The best-fitting model was then investigated for the modulation of the outcome variable by various fixed factors. The results showed that the complex model m2 yielded the lower AIC (AIC = 704.10) whereas the simpler model m1’s AIC was significantly higher (AIC = 781.78) [chi-square(6) = 81.68, *p* < 0.001].

**FIGURE 5:**
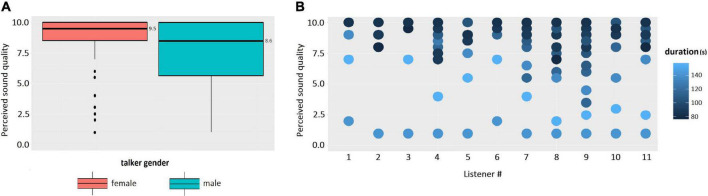
**(A)** Perceived sound quality vs. talker gender. **(B)** Perceived sound quality vs. duration of the spoken passage across two talker groups.

Speaking of the effects of the fixed factors on the perceived sound quality, the effect of TALKERGROUP was significant (beta = −0.857, *F*(1,162) = 6.89, *p* = 0.009), also the talker-group related differences in perceived sound quality in listeners can be observed in Figure 4, which demonstrates that the mean quality rating for the NH talker group ($\bar{x}$: 9.4, s.d: 0.8) was higher than that of the CI talker group ($\bar{x}$: 6.9, s.d: 3). A significant effect of the TALKERGENDER factor was also observed [beta = −0.094, *F*(1,162) = 10, *p* = 0.002] and Figure 5A visualizes the talker-gender related differences such that the median value of the perceived sound quality across the speech produced by female talkers (median = 9.5) was higher than that of the speech produced by male talkers (median = 8.6). Figure 5B visualizes the duration related difference in perceived sound quality across the listeners, and the effect of DURATION on the perceived sound quality was also statistically significant [beta = −0.973, *F*(1,162) = 92.9, *p* < 0.001]. In general, the perceived sound quality was higher for the speech of shorter duration of utterances.

### Envelope entrainment in response to speech spoken by cochlear implant talkers and normal-hearing talkers

An optimal regularization parameter λ that minimizes the MSE between the actual speech envelope and the reconstructed envelope was first selected to train the decoder, and followed by choosing the optimal integration window over which the decoder integrates the EEG to reconstruct the speech envelope. With these optimal parameters, the correlation between the actual speech envelope and the reconstructed envelope was computed as the metric to quantify the degree of cortical entrainment to the speech envelope. The computed metric is then compared between the two talker groups in relation to their perceived sound quality.

#### Entrainment to speech envelope (<40 Hz)

##### Selection of parameters to train decoders

An optimized λ was chosen from a set of values (10^–1^, 10^0^,…, 10^3^, 10^4^, 10^5^,.., 10^10^) which minimizes the MSE between the actual speech envelope and the reconstructed envelope as in Eq. 2. In both delta EEG band and theta EEG band, an optimal λ value was evaluated for each listener, and the value, however, turned out to be 10^6^ for all the listeners. In general, an integration window is chosen from the range of time lags between 0 and 400/500 ms (Crosse et al., 2016). Likewise, we varied the temporal integration window of the decoder from 0–20 ms to 0–400 ms with a step size of 20 ms and chose an integration window of 0–400 ms for all the listeners, as there was no consistent peaking of correlation vs. time lags was observed across spoken speech passages by CI talkers and NH talkers.

**FIGURE 6:**
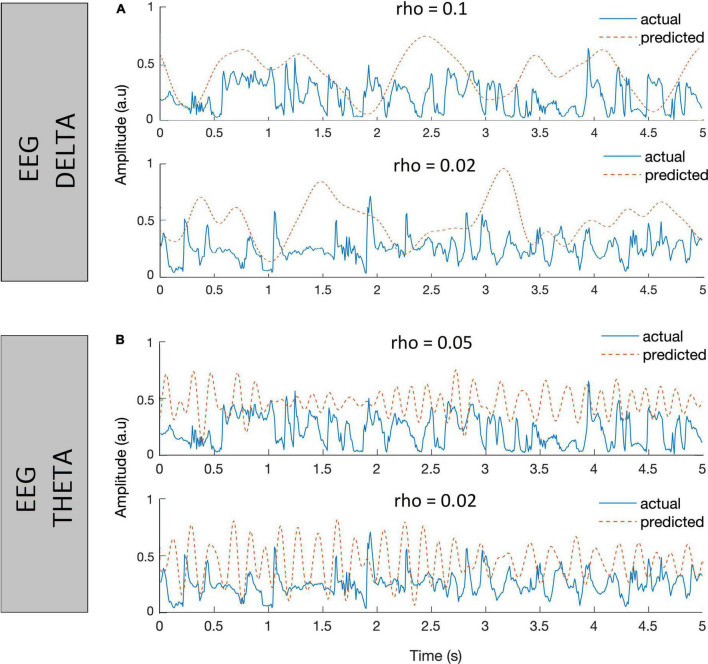
Plots showing instances of actual speech envelopes (blue) and the reconstructed envelopes (red dotted) and bootstrapped Spearman correlation (*rho*) between them. The decoders were trained using EEG in delta **(A)** and EEG in theta bands **(B)**, with speech envelope < 40 Hz.

The decoders are trained using these chosen parameters to reconstruct the envelope of speech from delta and theta EEG bands separately. Some instances of the reconstructed envelope and the actual speech envelope, and also the bootstrapped Spearman correlation “*rho*” between those two envelopes are shown in Figure 6 (Listener 6’s data). The correlation values shown at the top of the respective waveforms is considered as a metric reflecting the degree of cortical entrainment to the respective actual speech envelope. In general, a higher correlation value between the actual speech envelope and the reconstructed envelope indicates higher cortical entrainment to the actual speech envelope. Using the delta or theta EEG band and speech envelope < 40 Hz, the linear decoder poorly reconstructed the envelope from the EEG when correlated to the actual speech envelope. The highest value of correlation between the actual (for the speech passage spoken by NH talker #6) and predicted envelope observed was 0.1 using delta EEG band.

**FIGURE 7:**
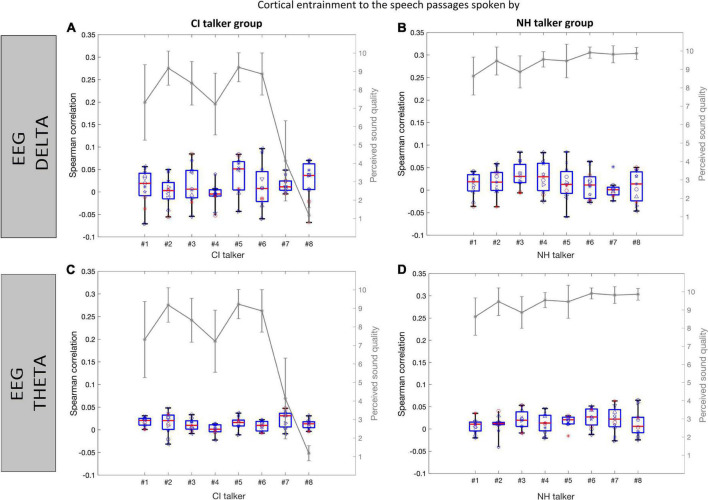
Cortical entrainment to speech envelope (<40 Hz) in the normal-hearing listeners is shown for each of the speech passages from the CI talker group **(A,C)** and NH talker group **(B,D)** using EEG in delta and EEG in theta bands, respectively. The data are represented in the form of the boxplots depicting medians (center mark) and interquartile ranges (the bottom and top edges of the box indicate the 25th and 75th percentiles). The boxplot shows the entrainment data variance across listeners, and the individual listener’s data points are plotted over it. Each listener is shown with a different symbol. The perceived sound quality curve (gray) is also shown for comparison.

Figure 7 presents the cortical entrainment to speech envelope (<40 Hz) with EEG in the delta (top panel) and theta (bottom panel) frequency bands for the speech passages spoken by two groups of talkers (CI and NH). The mean sound quality rating as assessed by normal-hearing listeners for each speech passage in the two talker groups is also shown over the entrainment boxplots to show their perceived sound quality. Overall, the range of correlation values computed between the speech envelopes and the reconstructed envelopes from the EEG was higher using the delta EEG band (Figures 7A,B) than those obtained using the theta EEG band (Figures 7C,D). The above observation was true for the two groups of talkers as well. Comparing the envelope entrainment between the two groups of talkers, a higher variation in the median values of the envelope entrainment among spoken speech passages within the CI talker group was observed as compared to the NH talker group using delta EEG band (Figures 7A,B). Similar to the sound quality ratings, the variability in the median value of the envelope entrainment among spoken speech passages is less obvious within the NH talker group using delta EEG band (Figure 7B). Also, the range of correlation values were reduced greatly from delta to theta bands in both talker groups and no difference could be observed between the two talker groups using the theta EEG frequency bands.

##### Statistical analysis

Statistical analyses was conducted to investigate whether there was a significant difference in envelope entrainment (correlation values) in response to the speech passages produced by the two talker groups. A paired-samples *t*-test was performed on the Spearman correlation values for the 11 listeners, each entry of the listener with a single averaged correlation value across CI talker group and NH talker group as two dependent samples in the delta and theta EEG bands separately. The *t*-test results showed no significant difference in the envelope entrainment between the two talker groups using the delta EEG band [*t*(10) = 0.28, *p* = 0.79] or using theta EEG band [*t*(10) = 1.9, *p* = 0.09]. Using delta EEG band, the mean value of the envelope entrainment across the NH talker group 0.17 and the mean value of envelope entrainment across the CI talker group was 0.15. Whereas, using the theta EEG band, the mean value of envelope entrainment across the NH talker group was 0.16 and that of the CI talker group was 0.13.

#### Entrainment to speech envelope filtered to match bandwidth of delta (0.5–4 Hz) and theta (4–8 Hz) bands

##### Selection of parameters to train decoders

**FIGURE 8:**
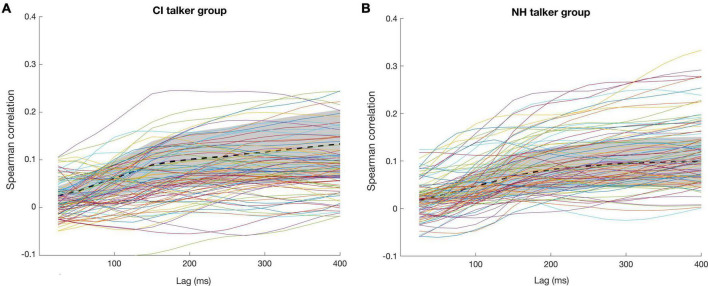
Spearman correlations between the actual speech envelope and the reconstructed envelope for **(A)** CI talker group and **(B)** NH talker group across the time lags. The thick dotted lines represent the mean values, and the shaded areas represent the standard deviation across the listeners.

**FIGURE 9:**
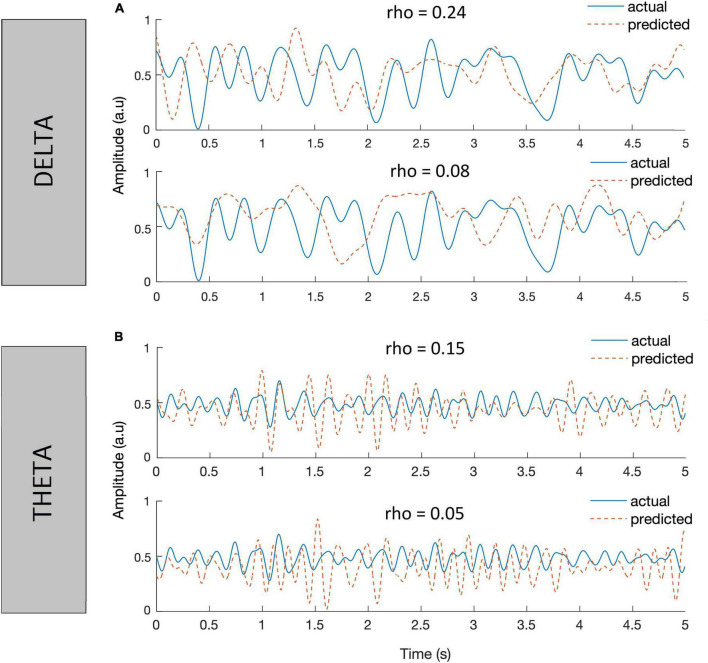
Plots showing instances of actual speech envelopes (blue) and the reconstructed envelopes (red dotted) and bootstrapped Spearman correlation (rho) between them in delta **(A)** and theta **(B)** bands.

For each listener, an optimal λ value was evaluated in both delta and theta bands and the values are tabulated in Table 3. Figure 8 presents the Spearman correlation as a function of different time lags in each talker group across 11 normal-hearing listeners (spoken speech passages by 8 talkers in each talker group*11 NH listeners = 8*11 curves). Overall, there was quite a large variability in the Spearman correlation curves across the time lags observed in both Figures 8A,B showing the inter-stimulus (talker) and inter-subject (listener) differences except for the earlier integration window of 0–150 ms. The earlier integration window of 0–150 ms resulted in comparatively lower correlation values across the listeners and the correlation values seem to increase as the upper bound of the time lag increases. Hence, the integration window to train a decoder was chosen as 150–400 ms across the NH listeners. The decoders are trained using these chosen parameters to reconstruct the envelope from the EEG in both delta and theta bands and some instances of improved correlation with the inclusion of band-pass filtered versions (delta and theta) of speech envelopes in the same listener shown previously (Figure 6) are presented in Figure 9.

**TABLE 3:** Individual best λ for NH listeners.

DELTA											
**Listener #**	**#1**	**#2**	**#3**	**#4**	**#5**	**#6**	**#7**	**#8**	**#9**	**#10**	**#11**
λ value	10^3^	10^2^	10^3^	10^3^	10^2^	10^2^	10^2^	10^3^	10^2^	10^2^	10^2^

**THETA**											

**Listener #**	**#1**	**#2**	**#3**	**#4**	**#5**	**#6**	**#7**	**#8**	**#9**	**#10**	**#11**

λ value	10^2^	10^2^	10^2^	10^2^	10^2^	10^2^	10^2^	10^2^	10^2^	10^2^	10^2^

**FIGURE 10:**
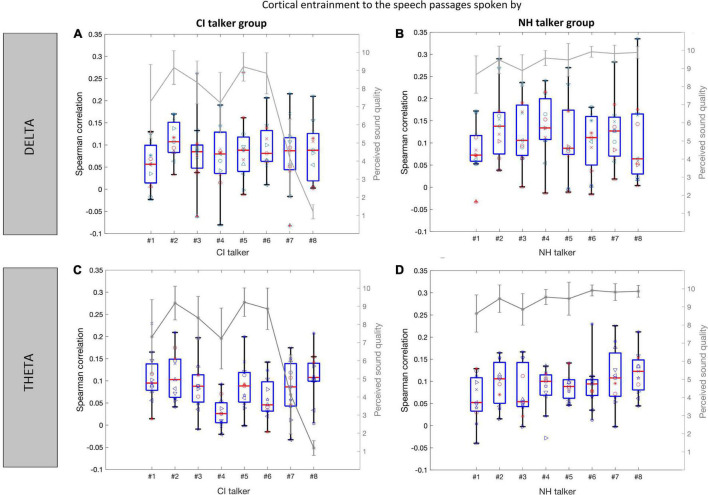
Cortical entrainment to speech envelope (filtered to match delta and theta bandwidth) in the normal-hearing listeners is shown for each of the speech passages from the CI talker group **(A,C)** and NH talker group **(B,D)** in delta and theta band, respectively. The data are represented in the form of the boxplots depicting medians (center mark) and interquartile ranges (the bottom and top edges of the box indicate the 25th and 75th percentiles). The boxplot shows the entrainment data variance across listeners, and the individual listener s data points are plotted over it. Each listener is shown with a different symbol. The perceived sound quality curve (gray) is also shown for comparison.

Figure 10 presents the envelope entrainment in response to the speech passages spoken by two groups of talkers (CI and NH), where the speech envelopes were also band-pass filtered into the delta (top panel) and theta (bottom panel) frequency bands. Overall, the range of correlation values observed in the delta band were broader (Figures 10A,B) compared to the range observed in the theta band (Figures 10C,D). Comparing the envelope entrainment between the two groups of talkers in the delta band, a higher variation in the median values of the envelope entrainment within the NH talker group (Figure 10B) as compared to the CI talker group (Figure 10A). In contrary with the results of entrainment to speech envelope (<40 Hz) (Figure 7A), the variability in the median value of the envelope entrainment is less obvious within the CI talker group in the delta band (Figure 10A). Looking at the behavioral sound quality, between the talker #1, #2, and #3 in both the talker groups, the speech passage spoken by talker #2 had a higher sound quality compared to that of talker #1 and #3. A similar trend was also observed in the entrainment data, i.e., median value of the envelope entrainment for the speech passage produced by talker #2 is higher compared to that of talker #1 and #3. The above observation was true in both delta and theta bands and in both groups of talkers. Compared to the decoder trained using the actual speech envelope (<40 Hz), the decoders trained using the speech envelopes filtered to match delta and theta EEG bandwidths were able to achieve a higher correlation and better quantify the difference observed from the speech passages spoken by CI talkers and NH talkers.

##### Statistical analysis

A paired-samples *t*-test was performed on the Spearman correlation values for the 11 NH listeners between the CI and NH talker groups in the delta and theta bands separately. In the theta band, the *t*-test did not reveal a significant difference in the envelope entrainment between the two talker groups [*t*(10) = 1.47, *p* = 0.17] that the mean value of envelope entrainment across the NH talker group was 0.09 and that of the CI talker group was 0.08. Whereas in the delta band, the *t*-test results showed a significant difference in the envelope entrainment between the two talker groups [*t*(10) = 3.2, *p* = 0.009]. The mean value of the envelope entrainment across the NH talker group ($\bar{x}$: 0.12) was significantly higher than the mean value of envelope entrainment across the CI talker group ($\bar{x}$: 0.08). Furthermore, in the delta band, we performed the similar lmer analysis presented before by replacing the outcome variable with Spearman correlation between the actual speech envelope and the reconstructed envelope. We assessed how the fixed factors TALKERGROUP, TALKERGENDER, and DURATION modulated Spearman correlations by fitting two separate hierarchical models: m1 and m2. Model comparison was carried out between the simpler model with the only fixed factor: TALKERGROUP, and the complex model comprising all the fixed factors: TALKERGROUP, TALKERGENDER, and DURATION. The ANOVA results show that there was no significant difference between the simpler model m1’s AIC (AIC = −533.29) and the AIC of the complex model m2 (AIC = −529.33) [chi-square(6) = 0.0318, *p* < 0.98]. The addition of the TALKERGENDER and DURATION fixed factors did not affect the goodness of fit of the model. With the simpler model m1, the lmer analysis results showed the TALKERGROUP had a significant effect on envelope entrainment [beta = −0.21, *F*(1,162) = 20.84, *p* < 0.001].

## Discussion

### Cochlear implant talker group vs. their speech quality

The database (Mendel et al., 2017) provided limited information about the individual CI talkers and this information is summarized in Table 4. As can be seen in Table 4 showing the demographic details of the chosen CI talkers, CI talkers #2, #5, and #6 were aided later than the others (marked in gray), presumably meaning a later onset of hearing loss. Therefore, better perceived speech quality likely reflects their greater degree of time in sound. The opposite occurred for CI talkers #7 and #8. These two talkers were aided earlier (marked in yellow), presumably indicating an earlier onset of hearing loss. Further, CI talker #8 used sign language only for communication whereas the others used oral communication and/or oral communication plus sign language. Their poorer speech quality likely reflects these factors.

**TABLE 4:** Demographic details of the selected CI talkers from the deaf speech corpus showing the mean sound quality rating of their spoken speech passages.

CI talker	Age (years)	Gender	Age of first amplification use (years)		Onset of hearing loss	Current type of amplification		Communication mode^4^	Mean perceived sound quality
			Right ear	Left ear		Right ear	Left ear		
#1	37	Male	5	5	Post-lingual	CI^2^	CI	Oral	7.3
#2	38	Female	28	NA^1^	Post-lingual	CI	NA	Oral	9.2
#3	16	Female	0.5	2	Pre-lingual	HA^3^	CI	Oral	8.4
#4	77	Male	18	73	Post-lingual	HA	CI	Oral and sign	7.2
#5	62	Female	38	38	Post-lingual	CI	HA	Oral and sign	9.2
#6	60	Male	52	52	Post-lingual	HA	CI	Oral and sign	8.9
#7	57	Female	3	58	Post-lingual	HA	CI	Oral	4.1
#8	33	Male	NA	3	Post-lingual	NA	CI	Sign only	1.2

^1^NA, not applicable. ^2^CI, cochlear implant. ^3^HA, hearing aid. ^4^Oral indicates that the talker used oral speech and language. Sign indicates that the talker used sign language. Gray highlight: Higher mean perceived sound quality. Yellow highlight: Lower mean perceived sound quality.

### Cortical entrainment to speech envelope

Correlation between the actual speech envelope and the reconstructed envelope was higher using the speech envelope filtered to match delta (0.5–4 Hz) and theta (4–8 Hz) bandwidth, when compared to the speech envelope with fluctuations < 40 Hz (Vanthornhout et al., 2018). In this scenario, between the delta and theta bands, in general, a closer cortical tracking to speech envelope was observed in the delta band. The observation was found in both talker groups. Previous study (Vanthornhout et al., 2018) has shown that cortical activity in delta band can serve as an indicator of how well a listener can recognize speech in the presence of noise. It is also known to carry the prosodic information (Goswami and Leong, 2013) to predict speech intelligibility (Ding and Simon, 2013; Vanthornhout et al., 2018). In alignment with the above studies, our results also suggest the cortical entrainment in delta band can serve as an indicator of the perceived sound quality by the listeners.

This preliminary research employed a linear model to predict and reconstruct the speech envelope from the EEG signal. The correlation values between the actual speech envelope and the reconstructed envelope obtained mainly ranged from −0.1 to 0.2. The reason for observing low correlation values between the two envelopes could be the assumption of a linear relationship between the speech envelope and the evoked neural response. A simple linear decoder is probably not a good fit to handle all the complexity of the auditory system and the brain (Vanthornhout et al., 2018). Also, neurons are known to respond to a complex stimulus like speech in a non-linear manner (Theunissen et al., 2000) and most likely, a non-linear decoder is needed to reconstruct the speech feature more accurately from the cortical responses. This idea is also supported by an EEG study conducted by Yang et al. (2015) which showed that compared with the linear regression model, the reconstructed spectrograms from the deep neural network achieved a higher average correlation with the actual spectrograms. Additionally, it is still not clear whether the envelope was a good representation of speech relevant to the perception of speech quality in normal-hearing listeners. Therefore, in the future, other speech features such as the spectrogram and phoneme-related features can be included as well in an attempt to improving the performance of a decoder (e.g., Di Liberto et al., 2015; Lesenfants et al., 2019a).

### Perceived sound quality vs. envelope entrainment to spoken speech passages by cochlear implant talkers and normal-hearing talkers

By looking at the results of behavioral sound quality ratings, we inferred that the higher average correlation between the actual speech envelope and the reconstructed envelope was found for the NH talker group who produced the speech with higher sound quality. This helped to prove our hypothesis that closer cortical tracking of speech envelope (higher correlation) was observed when speech was of higher perceived sound quality. However, we did not associate the sound quality ratings with the envelope entrainment results to find the relationship between them due to the difference in which the behavioral experiment was conducted compared to the EEG experiment. The behavioral data were collected when listeners were actively listening to speech passages, whereas EEG data were obtained when listeners were listening passively. Cortical entrainment has been seen in both active and passive paradigms of listening (for review, see Ding and Simon, 2014). A number of studies have shown that the cortical entrainment to speech is strongly modulated by attention and it has been shown that the reconstructed envelope depends strongly on the attentional focus of the listener and resembles the envelope of the attended speech (Kerlin et al., 2010; Ding and Simon, 2012). Hence, it is known that passive/active listening (Di Liberto et al., 2015; O’Sullivan et al., 2015) to speech affects the degree of cortical entrainment, and the difference in the two experimental setups would make it more difficult to extract appropriate conclusions about differences or similarities between behavioral and EEG results and this is one of the limitations of this study. In the future, the neural responses of the same normal hearing listeners using EEG while they paid attention to the speech passages can be recorded and how well the perceived sound quality ratings are correlated with the entrainment results can be analyzed. Our previous study (Akbarzadeh et al., 2021) has psychophysically validated the perceived sound quality measure with consistency to speech recognition scores and cortical entrainment outcome with normal hearing and hearing impaired listeners. The perceived sound quality measure adopted in the study is limited to the same perspective as previously studied. More work on the perceived sound quality measure will continue to cover a wider perspective of the measure.

## Conclusion

The present study shows that speech envelope is well-represented neurophysiologically. The speech envelope reconstructed from EEG using the regenerative model (Crosse et al., 2016) shows similarities to the actual speech envelope, particularly when the speech envelope is filtered to match the EEG bandwidth (delta and theta) of interest. Perceived sound quality ratings by the 11 normal-hearing listeners were found to be associated with the cortical activity involved in tracking the speech envelope. Closer tracking of the speech envelope, with higher correlations between the actual speech envelope and the envelope reconstructed from the EEG, was obtained in response to speech produced by NH talkers relative to CI talkers. Our results also show that the perceived sound quality differences rated by the normal-hearing listeners between speech passages spoken by CI talkers and NH talkers can be seen in the cortical tracking of the speech envelope in the same listeners.

## Data availability statement

The raw data supporting the conclusions of this article will be made available by the authors, without undue reservation.

## Ethics statement

The studies involving human participants were reviewed and approved by the Institutional Review Board at the University of Texas at Dallas. The patients/participants provided their written informed consent to participate in this study.

## Author contributions

SR, BM, SL, and C-TT were involved in conceptualization, data collection and analysis, and manuscript preparation. HC was involved in data collection. All authors contributed to the article and approved the submitted version.
